# Epidemiological investigation of the 119th confirmed Middle East Respiratory Syndrome coronavirus case with an indefinite mode of transmission during the Pyeongtaek outbreak in Korea

**DOI:** 10.4178/epih/e2015054

**Published:** 2015-12-10

**Authors:** Jong Hyuk Choi, Byoungin Yoo, Soon Young Lee, Eun Gyu Lee, Moran Ki, Woncheol Lee, Jong Rak Jung, Kyujin Chang

**Affiliations:** 1 Department of Preventive Medicine, Dankook University College of Medicine, Cheonan, Korea; 2 Department of Preventive Medicine, Soonchunhyang University College of Medicine, Cheonan, Korea; 3 Department of Preventive Medicine and Public Health, Ajou University School of Medicine, Suwon, Korea; 4 Division of Epidemiology and Health Index, Center for Genome Science, Korea Centers for Disease Control and Prevention, Cheongju, Korea; 5 Department of Cancer Control and Policy, Graduate School of Cancer Science and Policy, National Cancer Center, Goyang, Korea; 6 Epidemic Intelligence Service, Center for Infectious Disease Control, Korea Centers for Disease Control and Prevention, Cheongju, Korea; 7 Independent Health Consultant, Suwon, Korea

**Keywords:** Middle East Respiratory Syndrome coronavirus, Community-acquired infections, Communicable disease transmission, Disease outbreaks, Republic of Korea

## Abstract

Since the first case was diagnosed on May 20, 2015, there were 186 confirmed cases of Middle East Respiratory Syndrome (MERS) until the end of outbreak in South Korea. Although medical institutions were the most identifiable sources of MERS transmission in South Korea, similar to other countries, in-depth epidemiological investigation was required for some confirmed cases with indefinite contact history or hospital visit records. The subject of epidemiological investigation in the present study was a 35 year-old male patient diagnosed with MERS (#119) who lived in Asan-city and worked in Pyeongtaek-city. Various potential sources of transmission were carefully investigated. While he could have been exposed to MERS through a friend from Saudi Arabia or confirmed MERS cases in his workplace, neighboring areas, and medical institutions, as well as contacts in his home, the chances of transmission were low; however, the potential for transmission through his local community could not be excluded. Practically, it was difficult to determine the modes of transmission for all outbreak cases in communicable disease that occurred in this short period of time. The investigation to identify the mode of transmission in this case was ultimately unsuccessful. However, the various data collected and analyzed to reveal modes of transmission provided detailed information that could not be collected using only interview surveys.

## INTRODUCTION

Since the first outbreak in Saudi Arabia in 2012, about 1,600 patients worldwide were infected with Middle East Respiratory Syndrome (MERS) through the beginning of 2015 [1,2]. MERS is caused by infection with the MERS coronavirus (MERS-CoV). The virus can be transmitted between humans and animals and between humans. Transmission between humans mostly occurs in hospitals [3,4]. Although MERS-CoV may be transmitted within households, the risk is reportedly low [5-7].

Beginning with the first patient returning from the Middle East on May 11, 2015, there were 186 confirmed MERS patients and 38 deaths in South Korea (hereafter Korea) through the end of outbreak; most confirmed cases were nosocomial infections [8]. However, since some patients had indefinite dates of MERS symptom onset and contact history with confirmed MERS patients, in-depth epidemiological investigations were required to identify the likely sources of transmission. Among confirmed MERS patients whose transmission routes were unclear during the 2015 MERS outbreaks in Korea, this study investigated the potential sources of transmission of the 119th confirmed case, a 35-year-old man (#119), in order to prevent further spread of infection.

## MATERIALS AND METHODS

This in-depth epidemiological investigation was conducted by the central epidemiological investigation team of the Korea Centers for Disease Control and Prevention (KCDC), the epidemiological investigation team of Gyeonggi-do, and a private epidemiological investigation support team together with the epidemiological investigation team of community health centers in Pyeongtaek-city and Asan-city. The investigation methods included assessing the clinical progress and diagnosis of #119, tracing the route of transmission, and identifying and controlling the contacts.

Assessment of the clinical progress and diagnosis included review of hospital visit and medical records and an interview survey of #119. Case #119 was diagnosed with MERS based on positive test results for two MERS-CoV-specific genes (*upE/ORF1a*) by real-time reverse transcription polymerase chain reaction (real-time RT-PCR) or one MERS-CoV-specific gene (*ORF1b*) by conventional reverse transcription PCR (conventional RT-PCR) using sputum specimens. These tests were performed at the Chungnam Institute of Health and Environment and the KCDC. In addition, a serological test to measure antibody titers of MERS-CoV was used to assess MERS infection history.

For analysis of the potential modes of transmission, the chances of infection before May 31 (the suspected onset day of MERS symptoms) and the potential sources of exposure at the medical institutions he visited after May 31 were examined. In-depth investigation methods to trace the potential route of transmission in #119 utilized cellular phone location tracing data and credit card use records, closed-circuit television (CCTV) analysis, hospital visit records, medical records of each hospital, and interview surveys. Close contacts of #119 were identified and subjected to self-quarantine and/or active monitoring according to the MERS control manual of the KCDC, based on investigation data.

This investigation of private information was conducted for early detection of infected patients, infection prevention, and preservation of national health and safety based on Clause 2 of Article 76 in Law 13392 on the prevention and control of infectious diseases (request for information, etc.), and oral consent was obtained from all individuals. Since this national epidemiological MERS investigation was performed on an emergency basis in order to prevent large-scale outbreaks, it did not obtain pre-approval from the internal review board.

## RESULTS

### Clinical progress and diagnosis

**Figure 1.**
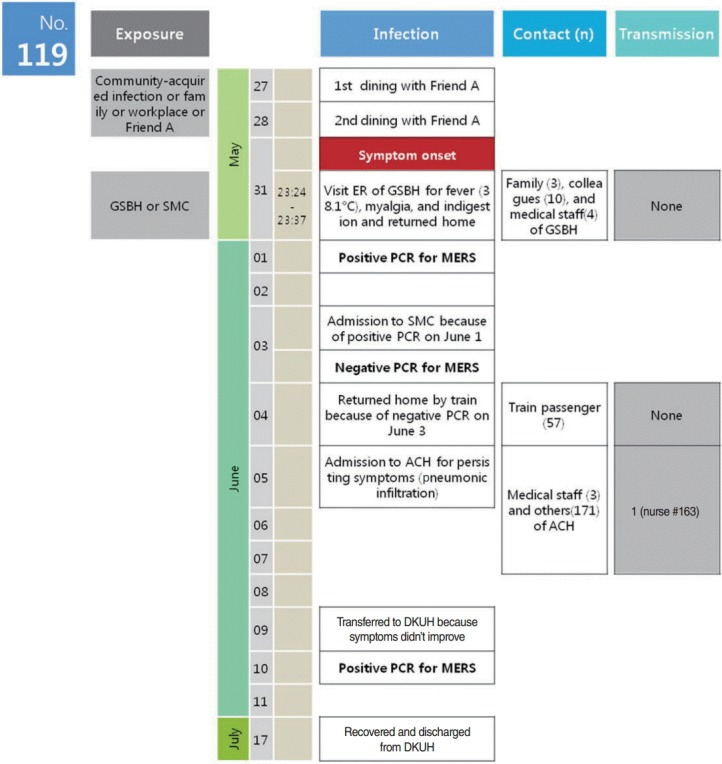
Chronology of major events and possible modes of transmission. PCR, Polymerase chain reaction test of sputum sample; The date of the PCR results is the sampling date; Friend A was a friend of #119 from Saudi Arabia; #163 is a nurse from ACH who had contact with #119; GSBH, Good Samaritan Bagae Hospital; SMC, Seoul Medical Center; ACH, Asan Chungmu Hospital; DKUH, Dankook University Hospital; MERS, Middle East Respiratory Syndrome.

Case #119 was a 35-year-old man working at a police station in Pyeongtaek, Gyeonggi-do, where the first MERS outbreak occurred in Korea. The patient lived in the Asan area, Chungcheongnam-do, a neighboring area of Pyeongtaek-city. Case #119 met Friend A, who had returned from Saudi Arabia, on May 27 and May 28. He ate sumac chicken for lunch on May 31, and developed symptoms including fever (38.1°C), hot flashes, myalgia, and dyspepsia; he visited the emergency room of the Good Samaritan Bagae Hospital (GSBH) around 11:00 pm on the same day, and returned home. GSBH suspected these symptoms to be MERS, and notified the Asan-city Community Health Center, which collected sputum specimens the next day (June 1) for testing at the Chungnam Institute of Health Environment. On June 3, based on the positive PCR test results of samples from #119, the patient was hospitalized in the isolation unit of the Seoul Medical Center (SMC). However, PCR tests performed on sputum specimens collected from #119 on June 3 (the day of admission to SMC) by the KCDC were negative, and he returned home on June 4. Nevertheless, he was hospitalized at Asan Chungmu Hospital (ACH) from June 5 to June 9 for persisting fever and chest discomfort, and was transferred to the Dankook University Hospital (DKUH) on June 9 due a lack of improvement of his pneumonia symptoms, where he was treated in the isolation unit. Sputum samples collected on June 10 by the KCDC tested positive by PCR, confirming that #119 was positive for MERS. Despite an exacerbation of acute respiratory distress syndrome, #119 was completely cured and discharged on July 19 (Figure 1).

### Suspected exposure events and transmission routes

#### Chances of infection before May 31

##### Friend A, who had returned from Saudi Arabia

Friend A complained of a sore throat on May 5 while working in Saudi Arabia; the friend returned to Korea on May 22 and had close contact with #119, having meals together on May 27 and May 28. Both #119 and Friend A were smokers, and reported in interview surveys that they had been smoking when they met on May 27 and May 28. Friend A visited ACH for a sore throat on June 1, and was prescribed antibiotics and anti-inflammatory analgesic drugs. After #119 was diagnosed with MERS based on positive sputum test results, Friend A also underwent sputum PCR testing on June 3 and June 11, both of which were negative. Friend A returned to Saudi Arabia on June 22 and showed no symptoms of infection afterward. In addition, a serological test on a blood sample collected from Friend A before leaving for Saudi Arabia was also negative.

##### Workplace exposure (Pyeongtaek Police Station)

Case #119 mostly worked inside the police station. His work area was about 23.1 m^2^ to 26.4 m^2^, and was shared by two investigation squads (about 10 people). In order to examine the possibility that #119 had come into contact with infected people in the work area, the list of people under investigation by the two investigation squads from the middle to the end of May was obtained and compared with the list of confirmed MERS patients and contacts with confirmed MERS patients, with no matches. In addition, interview surveys with #119 confirmed that he had no contact with people who had respiratory symptoms while working in the police station. There were no MERS patients among the other members of the investigation squads.

Additional investigations were performed to assess the risk of contact with confirmed MERS patients in places besides the police station, and in outdoor smoking areas other than the work area. Dates and times when #119 worked at the police station were identified and also compared to movements of eight confirmed MERS patients (#6, #13, #14, #15, #17, #18, #25, and #32) who could have visited the Pyeongtaek Police Station between May 17 and May 29, the suspected period of MERS transmission. However, none of these patients had visited the police station.

##### Local community exposure

###### - Local community exposure in Pyeongtaek-city

**Figure 2.**
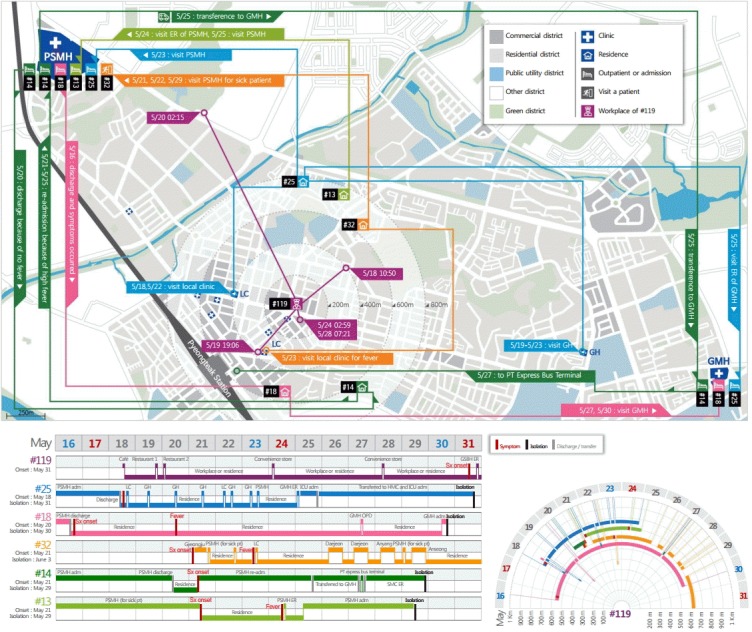
Spatiotemporal mapping of #119 and confirmed MERS patients suspected of transmitting their infections. Case #25 first experienced symptoms on May 15, and visited the local clinic and the geriatric hospital from May 18 to May 23. He visited the outpatient clinic at PSMH on May 23 and was admitted to GMH on May 25. Case #13 first experienced symptoms on May 21 and visited the ER of PSMH on May 24 for fever and other symptoms. He was admitted to PSMH on May 25. Case #32 experienced symptoms on May 21 and visited the PSMH clinic for sick patients on May 21 and May 22. He visited the local clinic with fever on May 23, and visited PSMH again on May 29. Case #14 was admitted to PSMH for pneumonia, but was discharged on May 20 because of no fever. The high fever recurred and the patient was re-admitted to PSMH on May 21 to May 25. He transferred to GMH on May 25 for persistent symptoms. He was discharged and visited the ER of SMC on May 27 because of no improvement. Case #18 was admitted to PSMH for pneumonia, but the symptoms were aggravated after discharge. He visited GMH on May 27 and May 30. Case #119 was in a nearby cafe on May 18, at 10:50. He was in the police station on May 19, 08:42, and in a restaurant on May 19, 19:06. He was in the police station on May 22 and 23 at 11:53 and 10:56. He also visited a convenience store on May 24, 02:59 and the police station on May 27, 13:16. He visited the convenience store again on May 28, 07:21. The possible exposure periods are the times between symptom onset and admission or isolation in each case. PSMH, Pyeongtaek St. Mary’s Hospital; GMH, Good Morning Hospital; SMC, Samsung Medical Center; LC, local clinic; ER, emergency room; GH, geriatric hospital; Sx, Symptom; adm, admission; pt, patient; PT, Pyeongteak.

The chances that #119 was exposed to confirmed MERS patients in the local community of Pyeongtaek-city between May 17 and May 29, focusing on restaurants and stores along the main street between the Pyeongtaek subway station and the Pyeongtaek Police Station, were also investigated. The movements of #119 were investigated in detail during this time period based on job records, credit card use records, and cellular phone location tracing data. According to the MERS epidemiological investigation report, 14 patients (#1, #6, #13, #14, #15, #16, #17, #18, #25, #32, #36, #75, #76, and #85) transmitted infections to at least one other person. These patients were considered to have high chances to cause community-acquired infections. Five of these confirmed MERS patients (#13, #14, #18, #25, and #32) were investigated because their possible periods of transmission overlapped with the suspected MERS exposure period of #119 and they were physically close to the main activity area of #119. Mapping based on the time of suspected transmission and spatial movement of #119 and the confirmed MERS patients found that no confirmed MERS patients spatially and temporally overlapped with #119 (Figure 2). Interviews revealed that #14 mostly traveled by car and rarely walked on the main street; similarly, #18 and #25 were expected to have limited outdoor activities due to severe infection symptoms between May 17 and May 29. Although #32 had visited a clinic located in a building next to a restaurant where #119 visited, the timing of visits did not overlap. In addition, on-site investigation performed based on the potential for #119 and #32 to meet by accident in the building revealed that the building had a structure that made it less likely for clinic traffic to overlap with that of neighboring restaurants.

###### - Local community exposure in Asan-city

Since there were several outbreaks of confirmed MERS patients in Asan-city, the residential area of #119, the chance of #119 coming into contact with confirmed MERS patients (#1 and #6) who lived in the Asan area or had activities in the area, such as visits to hospitals, was investigated. However, there was no overlap in residential areas or living zones (hospitals, workplaces, etc.) between #119 and other confirmed MERS patients, and #119 did not visit other areas in Asan area except for his residential area; he had not visited the clinics or hospitals, so there was no chance that he had had contact with other confirmed MERS patients.

##### Family

Case #119’s wife, a housewife, had no activity in areas other than Asan-city during the suspected exposure period, had no MERS-related symptoms, and had never visited any clinics or hospitals. However, a child of #119 visited a clinic in the Asan area for symptoms of upper respiratory infection on May 22 and May 23. The symptoms improved and the clinic had no visits by confirmed MERS patients before.

#### Chance of infection on and after May 31

##### The Good Samaritan Bagae Hospital

The chance of #119 having had contact with #22 or #52 in the emergency room of GSBH on May 31 was investigated. CCTV analysis showed that #119 visited the emergency room at 11:24 pm on May 31, left the emergency room at 11:37 pm on the same day and returned home. The responses to the interview survey indicated that he did not smoke while returning home. However, #52 visited the emergency room from 11:53 pm on May 31 to 03:16 am on June 1, whereas #22 was admitted through the outpatient department at 12:00 pm on May 30, was isolated, and was transferred to another hospital at 02:45 am on May 31.

##### Chance of infection at the Seoul Medical Center

The chances of infection from medical institutions after June 1, based on an assumption that the result of the sputum PCR test on June 1 was a false-positive, which possibility was very low, were investigated and described in detail in Appendix 1. The relation of three sputum PCR test results (June 1 to June 3) and onset date of MERS symptoms were also investigated in Appendices 2 and 3, which supported #119 was most likely infected before May 31. The all possible modes of transmission for #119 were summarized in Table 1.

### Identification and control of contacts

The close contacts of #119 (three family members and 10 workplace colleagues), four medical staff members in the emergency room of GSBH, and passengers on the train that he took home after discharge from the SMC were subjected to self-quarantine and/or active monitoring for 14 days from the last exposure day, and the medical staffs and patients of ACH were also subjected to cohort isolation in the hospital for 14 days from the last exposure day.

## DISCUSSION

This in-depth epidemiological investigation aimed to reveal the route of infection for confirmed MERS patient #119, who had an indefinite mode of transmission. The most likely onset date of MERS symptoms in #119 was May 31, corresponding to a suspected exposure date between May 17 and May 29. Although it was possible that #119 was infected with MERS during the meeting with Friend A on May 27 and May 28, after Friend A returned from Saudi Arabia, Friend A had no respiratory symptoms other than sore throat; two sputum PCR tests and a serological test were negative, so it was unlikely that #119 was infected by Friend A. The chances of #119 being infected in either his workplace in the Pyeongtaek Police Station or by his family in Asan-city were extremely low. Also, the chance that #119 had had contact with #22 or #55 in GSBH was extremely low. However, #119 had no record of clinic or hospital visits during the suspected exposure period, and confirmed MERS patients had moved around without knowing the facts of their own infection in Pyeongtaek-city and Asan-city, and then it was impossible to completely reconstruct the spatial and temporal movements of #119 and other confirmed MERS patients; thus, the chance of community-acquired infection could not be completely excluded. In addition, #119 had a relatively higher chance of contact with confirmed MERS patients in the local community of Pyeongtaek-city where there were more confirmed MERS patients than in Asan-city; thus, it is possible that #119 was infected in the main street near the Pyeongtaek Police Station, or the workplace. The area surrounding the police station corresponds to the downtown of Pyeongtaek-city, with many floating populations, and #119 often visited this area during off-hours, according to credit card use records and cellular phone location tracing data. However, his movements while his cellular phone and credit card were not in use were not identified, during which #119 might have had contact with confirmed MERS patients. Nevertheless, the temporal and spatial uncertainty made it impossible to determine where and with which patients #119 had contact.

In this case (#119) and other confirmed MERS patients with uncertain modes of transmission, it was difficult and often impracticable to trace each outbreak case of the communicable disease that prevailed in a short period time in order to clearly reveal the mode of transmission [9]. However, it is imperative to test various hypotheses while collecting and analyzing every possible data through on-site investigations that consider various possible modes of transmission. Finally, in cases of massive outbreaks of communicable disease, data acquisition systems are necessary to systematically collect various data in addition to interview surveys and professionals who can rapidly analyze the data and make decisions in order to more effectively identify modes of transmission and prevent transmission.

## Figures and Tables

**Table 1.** Summary of evidence for the possible modes of transmission to #119

Exposure	Possible modes of transmission (possible transmission periods)	Supporting evidence	Refuting evidence	Conclusion
Friend A of case #119	Friend A infected in Saudi Arabia and transmitted to #119 (May 27-May 28)	Friend A complained of sore throat on May 5 and returned from Saudi Arabia to Korea on May 22 Close contact with #119 in addition to smoking together on May 27 and May 28	No respiratory symptoms other than sore throat	Unlikely
			Negative results of two sputum PCR tests for MERS	
			Negative MERS antibody serological test results	
Pyeongtaek Police Station (#119’s workplace)	#119 infected in his workplace (May 17-May 29)	Within the incubation period	No confirmed MERS cases among the subjects investigated on the police team	Unlikely
			No MERS cases among his colleagues	
			No confirmed MERS cases visited the police station	
Pyeongtaek-city	#119 had community-acquired infection in Pyeongtaek-city (May 17-May 29)	Many MERS cases were in Pyeongtaek-city due to the outbreak in Pyeongtaek St. Mary’s Hospital	No confirmed MERS cases that spatially and temporally overlapped with #119	Possible
Asan-city	#119 had community-acquired infection in Asan-city (May 17-May 29)	Residence of #119 Confirmed MERS cases in Asan-city	No confirmed MERS cases that spatially and temporally overlapped with #119	Unlikely
Family of case #119	#119 infected from his family (May 17-May 29)	His children visited a clinic in Asan-city for upper respiratory symptoms	His children improved and no MERS cases had visited the clinic	Unlikely
			His wife had no respiratory symptoms	
GSBH	#119 infected in GSBH (May 31)	#22, #52 and #119 visited GSBH	#52 and #119 visited the ER of the hospital at different times	Unlikely
			#22 was isolated in a different room of the hospital when #119 visited	
			#119 did not smoke when he returned home	
SMC	#119 infected in SMC (June 3-June 4)	Assumed that the result of sputum PCR at June 1 was false positive	#119 was isolated in a different room from the other MERS cases	Unlikely
			Difficult to explain pneumonic infiltration on June 5	
			Low possibility of that the result of sputum PCR performed on June 1 was false-positive	

Friend A works in Saudi Arabia and returned to Korea on May 22. PCR, polymerase chain reaction; MERS, Middle East Respiratory Syndrome; GSBH, Good Samaritan Bagae Hospital; ER, emergency room; SMC, Seoul Medical Center.

## Appendix 1. Chance of infection at the Seoul Medical Center (SMC)

Based on an assumption that the result of the sputum polymerase chain reaction (PCR) test on June 1 was a false-positive, we investigated the possibility that #119 was infected at SMC, where he was hospitalized between June 3 and June 4, resulting in positive sputum PCR results on June 10. Re-inspection of the SMC found that no MERS patient was previously hospitalized in the lowpressure unit where #119 stayed, and he was completely isolated from four other confirmed MERS patients in terms of movements and areas visited during the hospitalization period.

In addition, if the findings of pneumonia at Asan Chungmu Hospital (ACH) on June 5 were assumed to be symptoms of MERS, the incubation period for #119 was 1 to 2 days. Moreover, since #119 was identified as having transmitted MERS to #163 at ACH between June 5 and 7, it is possible that this periods were transmittable symptomatic periods. Therefore, if it is assumed that the sputum PCR result from June 1 was a false-positive, then #119 was infected between June 3 and 4, and the date of symptom onset was between June 5 and 7; thus, the incubation period of #119 could be from 1 to 4 days. Considering that the point estimator of the median MERS incubation period is 6.83 days, with 5th and 95th percentiles of 2.27 and 13.48 days, respectively, the incubation period of #119 based on these assumptions is too short [A01]. In addition, since it is unusual for MERS to be transmitted immediately after onset of symptoms, the chance that #119 was infected at the SMC was extremely low.

## Appendix 2. Additional analysis on sputum PCR test results and onset date of MERS symptoms

The ranges of maximum exposure periods depending the possible sputum polymerase chain reaction (PCR) test results from June 1, 3, and 10 are summarized in Appendix 3. Hypotheses 1 and 2 in Appendix 3 are based on false positive sputum PCR results on June 1, while hypothesis 3 is based on a true positive sputum PCR test result on June 1. Hypotheses 1 and 2 indicate a chance of infection in the Seoul Medical Center. However, the chance of infection was extremely low for the reasons discussed in Appendix 1. In addition, #119 complained of fever and chest discomfort at Asan Chungmu Hospital, where he was hospitalized from June 5, and chest radiography revealed pneumonia in the right lung. Antibiotic treatment after his admission had no effect, with his fever persisting and pneumonia progressing; therefore, the onset date of his MERS symptoms were likely before June 5. Therefore, hypotheses 1 and 2 (based on false positive sputum PCR findings on June 1) were not the most likely explanation. Therefore, a true positive sputum PCR result on June 1, as assumed in hypothesis 3, is a more reasonable explanation for transmission between June 5 and 7. In conclusion, June 1 appears to be the most likely date for confirmation of the diagnosis.

Although #119 showed symptoms including fever, myalgia, and dyspepsia when he was admitted to Good Samaritan Bagae Hospital on May 31, he did not have urticaria; thus, his symptoms were more consistent with those of infection than with allergy due to consumption of sumac chicken. If June 1 is considered to be the date of confirmed diagnosis, it is reasonable to consider May 31 to be the date of symptom onset based on the clinical progress - infection, symptom onset, diagnosis.

## Appendix 3. Maximum exposure periods based on sputum polymerase chain reaction (PCR) test results

**Table:**

	Sampling date			Estimated exposure date
	June 1	June 3	June 10	
Sputum PCR results	Positive	Negative	Positive	
Hypothesis 1	False positive	False negative	True positive	June 1 - June 3
Hypothesis 2	False positive	True negative	True positive	June 3 - June 10
Hypothesis 3	True positive	False negative	True positive	May 18 - June 1
