# Supplementary material for: Epidemiological investigation of the 119th confirmed Middle East Respiratory Syndrome coronavirus case with an indefinite mode of transmission during the Pyeongtaek outbreak in Korea
Source: Epidemiol Health. 2015 Dec 10;37:e2015054. doi: 10.4178/epih/e2015054 (PMC4789606; doi:10.4178/epih/e2015054)
Supplement: Supplementary file 1 [file epih-37-e2015054-supplementary.pdf]

<제목표지>

(a) 제목(Title)

(국문) 평택에서 발생한 감염경로 미확인 119번째 메르스 확진자에 대한 역학조사

(b) 저자(Authors)

1저자: 최종혁[1], 유병인[2]

[1]단국대학교 의과대학 예방의학교실; [2]순천향대학교 예방의학교실 전공의

책임저자: 이순영[3]

[3]아주대학교 의과대학 예방의학교실

공저자: 이은규[4], 기모란[5], 이원철[6], 정종락[6], 장규진[7]

[4]질병관리본부 유전체센터 유전체역학과; [5]국립암센터 국제암대학원대학교 암관리정책학과; [6]질병관리본부 역학조사과; [7]보건 컨설턴트

(d) 책임저자 연락처

주소: Department of Preventive Medicine and Public Health, Ajou University School of Medicine, 164 World cup-ro, Yeongtong-gu, Suwon, Gyeonggi-do, 443-380, Repubic of Korea

전화: +82-31-219-5301

팩스번호: +82-31-219-5084

이메일 주소: solee5301@gmail.com

Running title: Transmission mode in the 119th confirmed MERS-CoV case

<초록(Abstract)>

한국에서는 2015 년 5 월 중동에서 유입된 첫 환자를 시작으로 하여 유행이 끝날 때까지 총 186 명의 메르스 확진자와 38 명의 사망자가 발생하였다. 한국에서의 메르스 전파는 다른 나라와 같이 대부분 의료기관 내에서 감염이 일어나는 것으로 파악되어 있으나, 일부 사례의 경우 확진 환자와의 접촉력이나 병원 방문 이력 등이 모호한 경우가 있어 이에 대한 심층적인 역학조사가 필요하였다. 본 연구의 역학조사대상은 아산시에 거주하며 평택시에 직장이 있는 35 세 남자 메르스 확진자 (#119)로, 감염경로에 대한 여러 가능성을 면밀히 검토하였다. 사우디아라비아에서 입국한 친구에 의한 메르스 노출 가능성, 직장 및 주변지역 그리고 의료기관에서 메르스 확진자로부터의 노출 가능성, 가정 내 접촉에 의한 가능성 등을 검토한 결과 각각의 가능성이 낮았으나 지역사회에서 전파되었을 가능성은 배제할 수 없었다. 단기간에 대규모로 발생한 감염병 유행의 모든 환례의 감염경로를 일일이 추적하여 명확하게 규명하는 것은 현실적으로 매우 어렵다. 이번 환자의 사례에서 감염경로 파악을 위해 여러 가능성을 검토하였으나, 최종적으로 감염경로를 규명하지 못한 한계점이 있다. 그러나, 감염경로 파악을 위해 다방면의 자료를 수집하고 분석함으로써 면접조사만으로 파악할 수 없었던 구체적인 정보를 통해 감염경로의 가능성을 체계적으로 검토할 수 있었다.

<주제어(Keywords)>

Middle East Respiratory Syndrome Coronavirus

Community-Acquired Infections

Communicable Disease Transmission

Disease Outbreaks

Republic of Korea

<본문(Manuscript Body)>

서론(Introduction)

중동호흡기증후군(Middle East Respiratory Syndrome, 이하 메르스)은 2012년 처음 사우디아라비아에서 환자가 발생한 이후 2015년 12월초까지 세계적으로 1600여명이 확진되었다 [1, 2]. 메르스는 메르스 코로나바이러스(Middle East respiratory syndrome coronavirus, MERS-CoV) 감염으로 발생한다. 이 바이러스는 동물과 사람, 사람과 사람 간에 전파되며, 사람과 사람 간 전파는 주로 병원 내에서 일어나는 것으로 알려져 있다 [3, 4]. 메르스 코로나바이러스의 전파는 가정 내에서도 일어날 수 있으나 가능성은 낮다고 알려져 있다 [5-7].

한국에서는 2015년 5월 11일 중동을 방문하고 온 첫 환자를 시작으로 유행이 끝날 때까지 186명의 메르스 확진자와 38명의 사망자가 발생하였으며, 대부분의 확진자가 병원 내 감염으로 밝혀졌다 [8]. 그러나 일부 사례는 환자의 메르스 증상발현일, 확진자와의 접촉력이 명확하지 않아 감염경로에 대한 심층적인 역학조사가 필요하였다. 이 조사는 2015년 한국 메르스 유행에서 감염경로가 명확하지 않은 확진자 중, 119번째로 확진된 35세 남자 환자(이하 #119, 이하 n번째로 메르스 확진된 환자를 #n으로 표시)의 감염경로를 파악하여 추가 감염 확산을 방지하기 위해 이루어 졌다.

방법(Methods)

이 조사는 평택시 및 아산시 보건소 역학조사반과 함께 질병관리본부 중앙역학조사반, 경기도역학조사반, 민간역학조사지원단이 심층 역학 조사한 결과이다. 조사 방법은 #119의 임상경과 및 진단, 감염경로 추적, 접촉자 파악 및 관리로 이루어 졌다.

임상경과 및 진단은 #119의 병원 방문기록 및 각 병원의 의무기록지 확인, 면접조사 등을 통하여 임상경과를 파악하였고, #119의 메르스 진단은 객담 검체에서 MERS-CoV 특정 유전자 2개(upE/ORF1a) Real-time Reverse Transcription Polymerase Chain Reaction (Real-time RT-PCR)가 양성이거나 MERS-CoV 특정 유전자 1개(ORF1b) Conventional Reverse Transcription Polymerase Chain Reaction (Conventional RT-PCR)가 양성일 때를 기준으로 이루어졌으며, 검사는 충청남도 보건환경연구원과 질병관리본부에서 시행하였다. 또한 메르스 과거 감염을 알아보기 위한 방법으로 MERS-CoV의 항체를 검사하는 혈청학적 검사를 이용하였다.

감염경로 추적은 #119의 메르스 증상 발현일로 추정되는 5월 31일을 기준으로 일반적으로 알려진 메르스 잠복기 (2~14일)에 따라 14일 전인 5월 17일부터 2일 전인 5월 29일 사이에 감염되었을 가능성과, 5월 31일 증상은 메르스 감염 증상이 아니라는 가정하에 그 이후에 검사와 입원을 했던 의료기관에서 감염되었을 가능성으로 나누어서 검토하였다. 구체적인 감염경로

조사방법은 #119의 휴대폰 위치추적, 신용카드 사용내역, CCTV 분석, 병원 방문기록 및 각 병원의 의무기록지 확인, 면접조사 등을 이용하였다.

접촉자 파악 및 관리는 #119의 역학조사를 바탕으로 질병관리본부 메르스 대응 지침에 따라 #119의 밀접접촉자를 설정하고, 자가격리 및 능동감시 조치를 취하였다.

이러한 개인 정보 등에 대한 조사는 감염환자의 조기 발견과 방역 및 국가의 보건 안전을 위해 법률 제 13392호 감염병의 예방 및 관리에 관한 법률 제 76조의 2(정보 제공 요청 등)에 따라 조사가 이루어 졌으며, 개인 동의는 구두 동의로 이루어 졌다. 이 조사는 국가가 대규모 유행을 방지하기 위해 시간을 다투며 긴급하게 실시하는 메르스 유행 역학조사로, 조사 이전에 별도의 기관심의위원회의 승인 절차는 수행되지 않았다.

결과(Results)

1. 임상경과 및 진단

#119는 한국에서 첫 MERS 유행이 발생한 경기도 평택의 경찰서에 근무하며 평택과 인접한 충청남도 아산지역에 거주하는 35세 남자이다. #119는 5월 27과 28일에 사우디아라비아에서 입국한 친구 A와 만났고 5월 31일 점심에 옻닭을 먹은 후 발열(38.1도), 열감, 근육통, 소화불량 등의 증상이 발생하여 당일 23시경 참좋은친구 박애병원 (The Good Samaritan Bagae Hospital, GSBH) 응급실에 내원하여 치료를 받고 귀가하였다. GSBH에서는 #119의 증상을 메르스로 인한 것으로 의심하여 아산시 보건소에 신고하였고 다음날인 6월 1일 아산시 보건소에서 객담 검체를 채취하여 충청남도 보건환경연구원으로 송부 하였다. #119는 6월 3일에 충청남도 보건환경연구원의 PCR 양성 판정을 받고 서울의료원 (Seoul Medical Center, SMC)에 격리 입원되었다. 그러나 6월 3일 SMC 입원 과정에서 채취한 #119의 객담 검체에 대하여 질병관리본부의 PCR 검사에서 음성 판정을 받고 6월 4일 자택으로 귀가하였다. 그러나 발열 및 흉부 불편감 등의 증상이 지속되어 6월 5일부터 6월 9일까지 아산충무병원 (Asan Chungmu Hospital, ACH)에 입원하였고, 폐렴증세 호전 없어 6월 9일 단국대학교병원(Dankook University Hospital, DKUH)으로 전원되어 격리 입원 치료를 받았으며 6월 10일에 채취한 객담 검체에서 질병관리본부의 PCR 양성판정으로 메르스가 확진 되었다. 이후 #119는 급성호흡곤란증후군(Acute respiratory distress syndrome)으로 악화되기도 하였으나 완치되어 7월 19일에 퇴원하였다 (Figure 1).

2. 감염경로 추정(suspected exposure events and transmission routes)

1) 5월 31일 이전 감염 가능성

① 사우디아라비아에서 귀국한 #119의 친구 (이하 친구 A)

친구 A는 사우디아라비아에서 근무하던 5월 5일경 인후통을 호소하였고 5월 22일 한국에 입국하여 5월 27일과 28일에 #119와 같이 식사를 하며 밀접 접촉을 가졌다. #119와 친구 A는 모두 흡연자로, 5월 27일과 28일 만났을 때에도 같이 담배를 피었다고 면접조사에서 응답하였다. 그 후 친구 A는 6월 1일에 인후통을 주소로 아산 충무병원에 내원하여 항생제 및 진통소염제를 처방 받았다. #119가 객담검사에서 메르스 양성 판정을 받은 후, 친구 A는 6월 3일과 6월 11일에 객담 PCR 검사를 받았으나 두 차례 모두 음성으로 판정되어 6월 22일에 사우디아라비아로 출국하였고 이후에도 감염증상을 보이지 않았다. 또한 친구 A가 사우디아라비아로 출국하기 전에 확보한 혈액검체의 혈청학적 검사에서도 음성으로 판정되었다.

② #119의 직장(평택 경찰서)에서 노출

#119는 주로 경찰서 내에서 근무하며, 근무 공간은 약 7~8평으로, 두 개의 수사팀(10여 명)이 공동으로 사용하고 있다. #119가 근무 공간에서 메르스 전파력이 있는 자와 접촉했을 가능성을 확인하기 위하여 5월 중순부터 5월말까지 #119와 같은 공간을 사용하는 두 팀의 수사대상자 명단을 확보하여 메르스 확진자 및 확진자와 접촉했던 자의 명단과 대조하였으나 일치하는 경우가 없음을 확인하였다. 또한 #119와의 면접조사결과 경찰서 근무 중 상기도 감염 증상을 호소하는 사람과 접촉하지 않았음을 확인하였다. 수사팀의 다른 근무자들에서 추가 메르스 환자가 발생하지도 않았다.

한편, 근무 공간이 아닌 경찰서 외부 흡연실 등에서 확진자와 마주쳤을 가능성을 추가적으로 조사하였다. #119가 경찰서에 근무했던 날짜와 시간을 조사하였으며 메르스 전파가 이루어졌을 것으로 예상되는 5월 17일부터 29일 사이에 평택에서 발생한 메르스 확진자로 평택경찰서 방문 가능성이 있는 확진자 8명 (#6, #13, #14, #15, #17, #18, #25, #32)의 경찰서 방문여부를 조사하였고, 8명 모두 방문하지 않았음을 확인하였다.

③ 지역사회에서 노출

-평택시 지역사회

5월 17일과 5월 29일 사이에 평택시 지역사회 (주로 평택 전철역에서 평택경찰서 사이의 번화가에 있는식당 및 상가)에서 메르스 확진자에 노출되었을 가능성에 대하여 조사하였다. 해당 기간 동안의 #119의 동선을 근무표, 신용카드 사용내역, 휴대폰 위치추적 등을 참조하여 세밀하게 파악하였다. 메르스 역학조사보고서를 검토하여, 지역사회 감염을 일으킬 가능성이 높은, 메르스를 1명 이상 다른 사람에게 전파한 환자 14명 (#1, #6, #13, #14, #15, #16, #17, #18, #25, #32, #36, #75, #76, #85) 중 그들의 전파가능 기간과 #119의 메르스 노출 의심 기간이 시간적으로 겹치며 #119의 주 활동지역과 공간적으로 가까운 메르스 확진자 5명 (#13, #14, #18, #25, #32)을

확인하였다. #119와 전파 의심 확진자들의 시간 및 공간적 움직임을 고려하여 맵핑 (mapping)을 해본 결과, #119와 시간 및 공간적으로 겹치는 메르스 확진자는 없었다 (Figure 2). #14는 본인의 자동차로 주로 이동하였으며 시내를 걸어 다닌 적은 거의 없다는 것을 면접을 통해 확인하였고, #18과 #25는 감염 증상이 심하여 5월 17일에서 29일 사이에 외부 활동을 거의 하지 못했을 것으로 확인되었다. #32의 경우 #119가 방문한 적이 있는 식당 옆 건물에 위치한 시내 의원을 방문한 적이 있지만, 시간적으로 겹치지 않았다. 또한 우연히 어느 시점에 #119가 #32와 그 건물에서 마주쳤을 가능성을 고려하여 현장 조사를 실시한 결과, 의원과 인근 식당들의 동선은 겹칠 가능성이 낮은 건물구조였다.

-아산시 지역사회

#119의 거주지역인 아산에서도 몇 명의 확진자들이 발생하였으므로, #119가 아산지역에 거주하거나 병원 방문 등 활동이 있었던 확진자들 (#1, #6)과 접촉하였을 가능성을 검토하였다. 그러나 #119와 다른 확진자들의 거주 및 생활권역(병원, 직장 등)이 겹치지 않았으며, #119는 아산지역에서 본인의 거주지 외 다른 지역을 방문하지 않았으며, 병·의원을 방문한 적도 없어 다른 확진자들과의 접촉가능성은 없었다.

④ 가족

#119의 처는 가정주부로 #119의 의심되는 노출 기간 동안 아산 외 지역에서의 활동이 없었고, 메르스 관련 증상도 없었으며, 병·의원을 방문한 적도 없었다. #119의 자녀는 상기도 감염 증상으로 5월 22일과 23일 아산 지역 의원을 방문하였으나 이후 증상은 호전되었으며, 해당 의원은 메르스 확진자가 경유한 적이 없는 곳이었다.

2) 5월 31일 이후 감염 가능성

① 참좋은친구 박애병원(GSBH)

#119가 5월 31일에 GSBH 응급실에서 #22, #52 와 접촉하였을 가능성을 조사하였다. CCTV 분석 결과 #119는 5월 31일 23시 24분에 응급실에 내원하여 치료 후 당일 23시 37분에 응급실에서 나와 귀가하였으며, 귀가 당시 흡연하지 않았음을 면접조사로 확인하였다. 한편 #52는 5월 31일 23시 53분에서 6월 1일 03시16분 사이에 응급실을 이용하였으며, #22는 5월 30일 12시 00분에 외래경유 내원하여 입원하였으나 격리되어 있다가 5월 31일 02시 45분에 다른 병원으로 전원 되었다.

② 서울의료원에서의 감염 가능성에 대한 조사

#119의 6월 1일 객담 PCR 결과가 위양성이라 가정하에, 6월 3일과 6월 4일 사이에 입원했던 서울의료원에서 감염되었을 가능성에 대해 검토하였으나, 가능성이 매우 낮은 것으로 판단되었다 (별첨 1). 또한 6월 1일, 6월 3일, 6월 10일 객담 PCR 검사 결과와 메르스 증상 발현일을 검토해 볼 때 6월 1일 이후 감염 가능성은 매우 낮았다 (별첨 2,3). #119 환자의 감염경로 가능성에 대한 검토결과는 표 1에 제시하였다.

3. 접촉자 파악 및 관리

#119의 밀접접촉자 등 (가족 3명, 직장 사무실 동료 등 10명), GSBH 응급실 의료진 등 4명, SMC 퇴원 후 귀가 시 이용했던 열차 탑승객에 대하여 각 최종노출일로부터 14일간 자가격리 및 능동감시 조치를 취하였고, 아산충무병원 의료진 및 환자에 대하여 최종노출일로부터 14일간 병원 내 코호트 격리 조치를 실시하였다.

토론(Discussion)

감염경로가 불명확한 메르스 확진자 #119에 대한 심층역학조사를 통하여 감염 경로를 밝혀내고자 하였다. #119의 메르스 증상 발현일은 5월 31일로 보는 것이 합리적이며 따라서 노출일은 5월 17일부터 5월 29일 사이일 것으로 추정된다. #119가 사우디아라비아에서 귀국한 친구A를 5월 27일과 28일에 만났을 때 메르스에 감염되었을 가능성을 검토하였으나, 친구 A는 인후통 증상 이외에는 호흡기 증상을 호소하지 않았으며, 두차례 객담 PCR 검사 및 혈청학적 검사에서 모두 음성으로 판정되었기 때문에, 친구 A로부터 감염 되었다고 보기는 어려웠다. #119의 직장인 평택경찰서와 아산의 가족에 의한 감염 가능성은 매우 낮은 것으로 평가되었다. 또한 #119가 #22, #55 와 GSBH 에서 접촉했을 가능성은 매우 낮다. 그러나 의심되는 노출기간 동안 #119는 병·의원 방문력이 없고, 평택과 아산에는 메르스 확진자들이 감염 사실을 모른 채 지역내 의료기관을 옮겨 다니고 있었으며, #119와 다른 메르스 확진자들의 동선을 시간 및 공간적으로 완전하게 그려내는 것은 불가능하기 때문에 지역사회 감염 가능성을 배제할 수는 없었다. 또한 아산시와 평택시 중에서는 메르스 확진자가 많았던 평택시 지역사회에서 접촉이 일어났을 가능성이 상대적으로 높으며 근무지인 평택경찰서 근처의 번화가가 가능성이 있다. 경찰서 주변은 평택시의 중심가에 해당하며 많은 유동인구가 이용하는 곳으로 신용카드 사용내역 및 휴대폰 위치추적상 #119가 근무 외 시간에 이 지역을 자주 방문하였던 것을 확인하였다. 그러나 휴대폰 및 신용카드를 사용하지 않은 기간의 동선은 확인되지 않았으며, 이러한 누락된 기간 동안 메르스 확진자와 접촉이 일어 났을 가능성이 있다. 다만, 정확히 어떤 환자와 어디서 접촉하여 감염되었는지를 시간 및 공간적으로 완전하게 그려낼 수 없는 불확정성 때문에 단언하기는 어렵다.

#119 이외에도 감염경로가 불명확한 다른 메르스 확진자의 경우와 같이, 감염병 유행이 단기간에 대규모로 발생할 때 모든 환례의 감염경로를 일일이 추적하여 명확하게 규명하는 것은 현실적으로 매우 어렵다 [9]. 그러나 다양한 감염경로의 가능성을 열어 두고 현장조사를 통하여 다방면으로 가능한 자료를 수집, 분석 하면서 여러 가설 등을 검증해보는 것은 매우 중요하다. 감염병 대유행시 감염경로를 더욱 효율적으로 명확하게 파악하고 전파를 빠르게 차단하기 위해서는 면접조사뿐만 아니라 다방면의 자료들을 체계적으로 수집할 수 있는 자료 확보체계와 이를 신속히 분석하고 판단할 수 있는 전문 인력이 뒷받침되어야 할 것이다.

<감사의 글(Acknowledgements)>

이번 역학조사에 많은 도움을 주신 평택시 보건소(소장 정병성), 송탄시 보건소(소장 양희종) 및 병원 관계자 분들께 진심으로 감사의 말씀을 드린다. 특히 초기 역학조사 자료를 제공하고 논문 자료 활용을 허락하여 주신 질병관리본부 및 각 시도 역학조사관 제위께 깊은 감사를 드린다. 무엇보다도 확진자로 치료 받으시는 어려운 중에도 역학조사에 적극 협조해주신 #119 환자분과, 가족·동료·지인들, 관련자로 추가 조사를 받으셔야 했던 친구 A와 가족들, 다른 확진자들 등 질병으로도 고통 받으시고 추가 조사로 인해 더욱 괴로움을 겪으신 모든 분들께 고개 숙여 깊은 위로의 말씀을 드린다.

<표와 그림(Tables and Figures)>

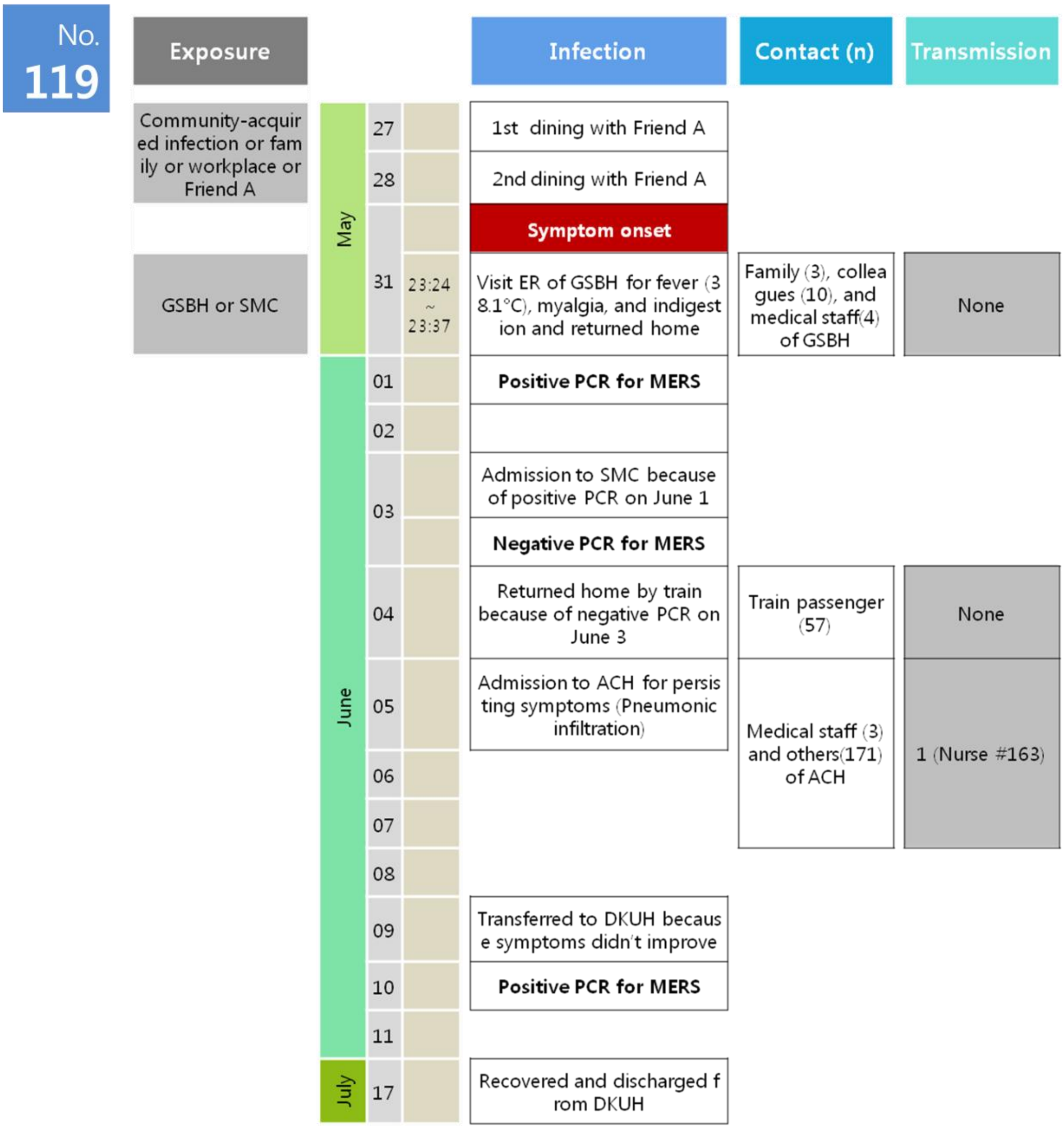

그림 1. 시간대별 중요 사건들과 가능한 전파 경로.

Figure 1. Chronology of major events and possible modes of transmission. PCR, Polymerase chain reaction test of sputum sample; The date of the PCR results is the sampling date; Friend A was a friend of #119 from Saudi Arabia; #163 is a nurse from ACH who had contact with #119; GSBH, Good Samaritan Bagae Hospital; SMC, Seoul Medical Center; ACH, Asan Chungmu Hospital; DKUH, Dankook University Hospital; MERS, Middle East Respiratory Syndrome.

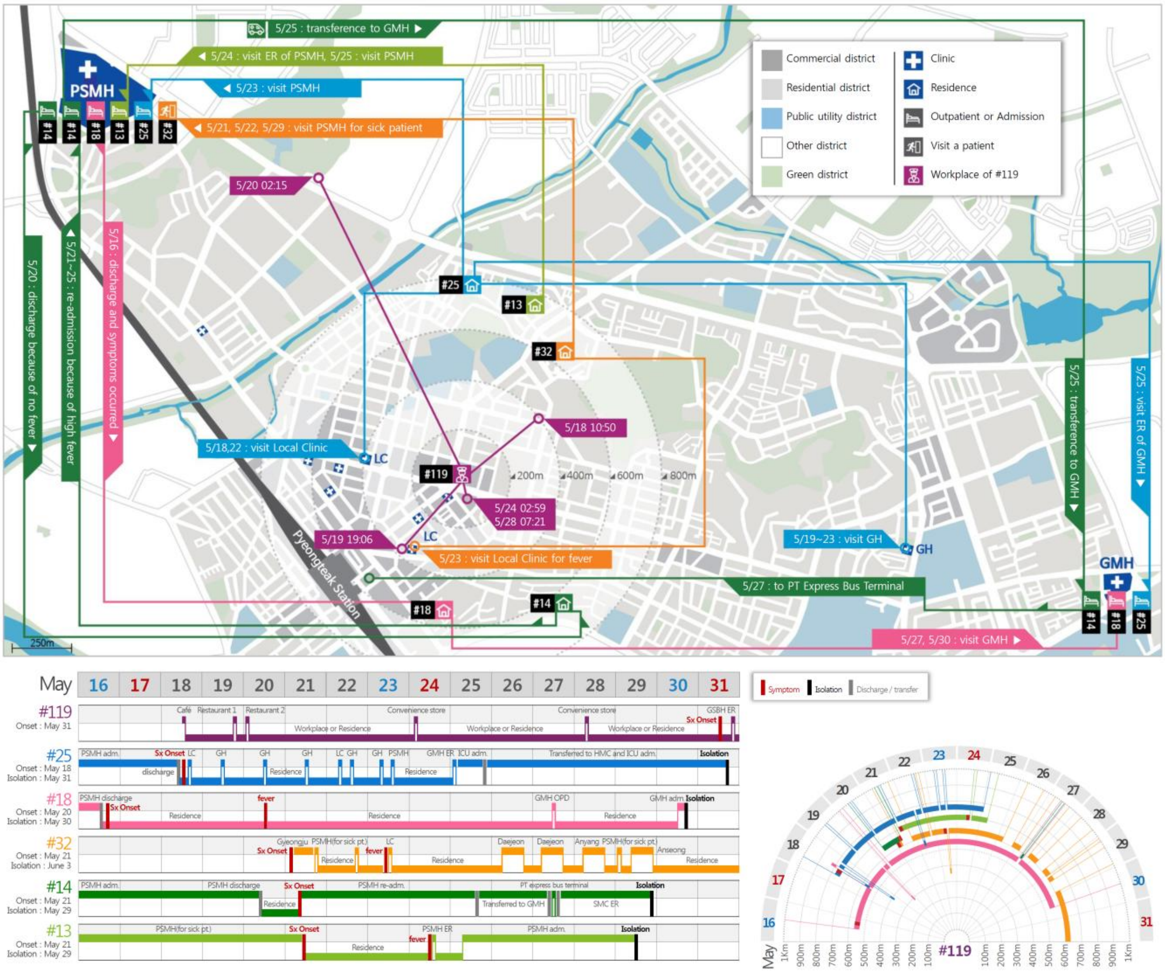

그림 2. 전파 의심 확진자들과 #119의 시간-공간적 맵핑

Figure 2. Spatiotemporal mapping of #119 and confirmed MERS patients suspected of transmitting their infections. Case #25 first experienced symptoms on May 15, and visited the local clinic and the geriatric hospital from May 18–May 23. He visited the outpatient clinic at PSMH on May 23 and was admitted to GMH on May 25. Case #13 first experienced symptoms on May 21 and visited the ER of PSMH on May 24 for fever and other symptoms. He was admitted to PSMH on May 25. Case #32 experienced symptoms on May 21 and visited the PSMH clinic for sick patients on May 21 and 22. He visited the local clinic with fever on May 23, and visited PSMH again on May 29. Case #14 was admitted to PSMH for pneumonia, but was discharged on May 20 because of no fever. The high fever recurred and the patient was re-admitted to PSMH on May 21–May 25. He transferred to GMH on May 25 for persistent symptoms. He was discharged and visited the ER of SMC on May 27 because of no improvement. Case #18 was admitted to PSMH for pneumonia, but the symptoms were aggravated after discharge. He visited GMH on May 27 and 30. Case #119 was in a nearby cafe on May 18, at 10:50. He was in the police station on May 19, 08:42, and in a restaurant on May 19, 19:06. He was in the police station on May 22 and 23 at 11:53 and 10:56. He also visited a convenience store on May 24, 02:59 and the police station on May 27, 13:16. He visited the convenience store again on May 28, 07:21. The possible exposure periods are the times between symptom onset and admission or isolation in each case. PSMH, Pyeongtaek St. Mary's Hospital; GMH, Good Morning Hospital; SMC, Samsung Medical Center; LC, local clinic; ER, emergency room; GH, geriatric hospital; Sx, Symptom; adm., admission; pt., patient; PT, Pyeongteak.

표 1. 가능한 전파경로의 근거 요약 (Table 1. Summary of the evidence of possible modes of transmission from others to #119)

Exposure	Possible modes of transmission (Possible transmission periods)	Supporting evidence	Refuting evidence	Conclusion
Friend A of case #119	Friend A infected in Saudi Arabia and transmitted to #119 (5/27, 5/28)	Friend A complained of sore throat on May 5 and returned from Saudi Arabia to Korea on May 22<br><br>Close contact with #119 in addition to smoking together on May 27 and 28	No respiratory symptoms other than sore throat<br><br>Negative results of two sputum PCR tests for MERS<br><br>Negative MERS antibody serological test results	Unlikely
Pyeongtaek Police Station (#119's workplace)	#119 infected in his workplace (5/17-5/29)	Within the incubation period	No confirmed MERS cases among the subjects investigated on the police team<br><br>No MERS cases among his colleagues<br><br>No confirmed MERS cases visited the police station	Unlikely
Pyeongtaek city	#119 had community-acquired infection in Pyeongtaek city (5/17-5/29)	Many MERS cases were in Pyeongtaek city due to the outbreak in Pyeongtaek St. Mary's Hospital	No confirmed MERS cases that spatially and temporally overlapped with #119	Possible

Asan city	#119 had community-acquired infection in Asan city (5/17-5/29)	Residence of #119<br><br>Confirmed MERS cases in Asan city	No confirmed MERS cases that spatially and temporally overlapped with #119	Unlikely
Family of case #119	#119 infected from his family (5/17-5/29)	His children visited a clinic in Asan city for upper respiratory symptoms	His children improved and no MERS cases had visited the clinic<br><br>His wife had no respiratory symptoms	Unlikely
GSBH	#119 infected in GSBH (5/31)	#22, #52 and #119 visited GSBH	#52 and #119 visited the ER of the hospital at different times<br><br>#22 was isolated in a different room of the hospital when #119 visited<br><br>#119 did not smoke when he returned home	Unlikely
SMC	#119 infected in SMC (6/3-6/4)	Assumed that the result of sputum PCR at Jun. 1 was false positive	#119 was isolated in a different room from the other MERS cases<br><br>Difficult to explain pneumonic infiltration on June 5<br><br>Low possibility of that the result of sputum PCR performed on June 1 was false-positive	Unlikely

Friend A works in Saudi Arabia and returned to Korea on May 22. PCR, polymerase chain reaction; MERS, Middle East Respiratory Syndrome; GSBH, Good Samaritan Bagae Hospital; ER, emergency room; SMC, Seoul Medical Center.

<참고문헌(Reference)>

1. World Health Organization (WHO). Middle East respiratory syndrome coronavirus (MERS-CoV) Summary of Current Situation, Literature Update and Risk Assessment. 2015. Available from http://www.who.int/csr/disease/coronavirus_infections/risk-assessment-7july2015/en/.
2. Centers for Disease Control and Prevention (CDC). Severe respiratory illness associated with a novel coronavirus--Saudi Arabia and Qatar, 2012. MMWR Morb Mortal Wkly Rep 2012;61:820.
3. Zumla A, Hui DS, Perlman S. Middle East respiratory syndrome. Lancet 2015;386:995-1007.
4. Oboho IK, Tomczyk SM, Al-Asmari AM, Banjar AA, Al-Mugti H, Aloraini MS, et al. 2014 MERS-CoV outbreak in Jeddah--a link to health care facilities. N Engl J Med 2015;372:846-854.
5. Memish ZA, Zumla AI, Al-Hakeem RF, Al-Rabeeah AA, Stephens GM. Family cluster of Middle East respiratory syndrome coronavirus infections. N Engl J Med 2013;368:2487-2494.
6. Drosten C, Meyer B, Muller MA, Corman VM, Al-Masri M, Hossain R, et al. Transmission of MERS-coronavirus in household contacts. N Engl J Med 2014;371:828-835.
7. Abroug F, Slim A, Ouanes-Besbes L, Hadj Kacem MA, Dachraoui F, Ouanes I, et al. Family cluster of Middle East respiratory syndrome coronavirus infections, Tunisia, 2013. Emerg Infect Dis 2014;20:1527-1530.
8. Ki M. 2015 MERS outbreak in Korea: hospital-to-hospital transmission. Epidemiol Health 2015;37:e2015033.
9. Chang K, Ki M, Lee EG, Lee SY, Yoo B, Choi JH. MERS epidemiological investigation to detect potential mode of transmission in the 178th MERS confirmed case in Pyeongtaek, Korea. Epidemiol Health 2015;37:e2015036.

<별첨 1 (Appendix 1)>

서울의료원에서의 감염 가능성에 대한 조사

#119의 6월 1일 객담 PCR 결과가 위양성이라 가정하고, 6월 3일과 6월 4일 사이에 입원했던 서울의료원에서 감염되어 6월 10일 객담 PCR 양성 판정으로 확진 되었을 가능성을 검토하였다. 서울의료원을 재조사한 결과, #119가 입원했던 음압 병실에는 그 이전에 메르스 환자가 입원하지 않았으며, 입원 중 다른 메르스 확진자 4명과 동선 및 공간이 완전히 격리되어 있었던 것으로 확인되었다.

또한 #119는 6월 5일 아산충무병원 입원 당시 폐렴소견을 보였기 때문에, 이를 메르스 증상이라고 가정하면 잠복기는 1일에서 2일이다. 또한 #119는 아산충무병원에서 6월 5일에서 6월 7일 사이에 #163에게 메르스를 전파시킨 것으로 확인 되었으므로, 이 기간은 전파 가능한 증상시기라고 할수있다. 따라서 6월 1일 객담 PCR 결과가 위양성이고 6월 3일과 6월 4일 사이에 감염 되었고 증상 발생을 6월 5일에서 7일이라고 하면, #119의 잠복기는 최소 1일에서 최대 4일이다. 메르스 잠복기 중위수의 점추정량은 6.83일, 5 백분위수는 2.27일, 95 백분위수는 13.48일인 것으로 볼 때, 이 가정에서의 #119의 잠복기는 짧은 편이다 [A01]. 또한 증상 발생 직후에 메르스를 전파시키기는 어렵다는 점을 고려하면 #119가 서울의료원에서 감염되었을 가능성은 매우 낮은 것으로 판단된다.

A01. Korea Centers for Disease Control and Prevention. Middle East Respiratory Syndrome Coronavirus Outbreak in the Republic of Korea, 2015. Osong Public Health Res Perspect 2015;6:269-278.

<별첨 2 (Appendix 2)>

객담 PCR 검사 결과 및 메르스 증상 발현일에 대한 추가 분석

6월 1일, 6월 3일, 6월 10일 객담 PCR 검사 결과에 대한 참, 거짓 여부에 따른 최대한의 노출 기간의 범위를 정리하면 별첨3의 표와 같다. 별첨3의 표의 가정 1, 가정 2는 6월 1일 객담 PCR 검사 결과가 위양성일 경우이고, 가정 3은 6월 1일 객담 PCR 검사 결과가 실제 양성일 경우이다. 가정 1, 가정 2의 경우는 서울의료원에서의 감염 가능성을 생각할 수 있다. 그러나 이 가능성은 위에서 검토한 바와 같이(별첨1), 가능성이 낮다. 또한 6월 5일부터 입원했던 아산충무병원에서 #119는 발열 및 흉부 불편감을 호소하였고 흉부 방사선 촬영상 우측 폐에 폐렴소견을 보였으며 입원 후 시행한 항생제 치료에 반응하지 않고 지속적으로 열이 나고 폐렴이 진행된 것을 근거로 6월 5일 이전에 이미 메르스 증상이 발현되었을 것으로 보여 가정 1, 가정 2 (6월 1일 객담 PCR 위양성 가정)는 가능성이 낮다. 따라서 가정 3과 같이 6월 1일 객담 PCR 검사 결과를 실제

양성으로 보았을 때 6월 5일-7일 사이 감염 전파를 설명하기에 더욱 적절하다. 따라서 6월 1일을 확진일로 삼는 것은 어느 정도 합리적이다.

메르스 증상 발현일에 대하여 #119는 5월 31일 박애병원 내원 당시 발열 및 근육통, 소화불량 등의 증상이 있었으나 두드러기 증상을 보이지 않아 옻닭으로 인한 알레르기 증상이라기보다는 감염 증상에 더 가깝다는 것을 확인하였다. 6월 1일을 확진일로 본다면, 감염, 증상발현, 진단의 임상 경과로 볼 때 5월 31일을 증상 발현일로 삼는 것은 합리적이다.

<별첨 3 (Appendix 3)>

객담 PCR 결과의 참 거짓 여부와 그에 따른 최대한의 노출 날짜의 범위

	Sampling date			Estimated exposure date
	**June 1**	**June 3**	**June 10**	
**Sputum PCR results**	Positive	Negative	Positive	
**Hypothesis 1**	False positive	False negative	True positive	June 1 - June 3
**Hypothesis 2**	False positive	True negative	True positive	June 3 - June 10
**Hypothesis 3**	True positive	False negative	True positive	May 18 - June 1
